# Re: Evaluation of microneedling with and without injectable-platelet rich fibrin for gingival augmentation in thin gingival phenotype—A randomized clinical trial

**DOI:** 10.1016/j.jobcr.2024.03.004

**Published:** 2024-05-22

**Authors:** Chetana, Sangamithra Sidharthan, Gopalakrishnan Dharmarajan, Shraddha Iyer, Mariam Poulose, Meghana Guruprasad, Dhakshay Chordia

**Affiliations:** Department of Periodontology, Dr. D.Y. Patil Dental College and Hospital, Dr. D.Y. Patil Vidyapeeth, Sant Tukaram Nagar, Pimpri, Pune, 411 018, Maharashtra, India; Department of General Surgery, Saveetha Medical college Hospital, Thandalam, Chennai, 602105, India

Greetings, Esteemed Editor,

We express our gratitude for the chance to address the concerns expressed in the letter from Dr. Carlos Fernando Mourão et al. and to provide further information about our blinding, comparison groups, and conclusions in consideration of these issues. We thank Dr. Carlos Fernando Mourão and the team for sharing their concerns and showing interest in our paper. We intend to thoroughly address all doubts and concerns raised.

Firstly, we wish to elucidate that the assignment of particular treatments to the procedure site (Microneedling/Microneedling + i-PRF) was pre-established via a computer-generated random sequence. When we mentioned blinding participants, we intended that participants underwent both treatments, with only the site receiving the additional i-PRF injection being undisclosed. We apologize for any ambiguity or lack of clarity surrounding this issue.

Addressing the next query, the significance of the placebo or sham treatment group in randomized clinical trials cannot be overlooked or underestimated, as it greatly enhances the credibility of the evidence produced. Any, favorable or unfavorable outcomes in the control group may indeed be influenced by the placebo effect. However, it's important not to dismiss the potential therapeutic impact of administering saline^1^^,^^2^ or another inert substance at the treatment site, especially considering the regimen involving four sessions. Consequently, such administration was avoided in our study.

Regarding the question about multiple injections in the thin gingiva, we'd like to clarify that the maximum amount of i-PRF injected was 3 ml, with 2–3 injections per site aimed at achieving fullness. Moreover, the injections targeted the keratinized tissue region, avoiding the free marginal tissue to mitigate any untoward incidence.

We want to emphasize that we have explicitly stated the necessity for a longer follow-up period, histological evaluation, and a larger sample size to ascertain the validity of this initial data. Nonetheless, in this specific trial, the utilization of i-PRF indeed augmented the beneficial impact of microneedling.

## Source(s) of support

Nil.

## Declaration of competing interest (If present, give more details)

Nil.
